# Evaluation of the Constant score: which is the method to assess the objective strength?

**DOI:** 10.1186/s12891-019-2795-6

**Published:** 2019-09-04

**Authors:** Patrick Ziegler, Luise Kühle, Ulrich Stöckle, Elke Wintermeyer, Laura E. Stollhof, Christoph Ihle, Christian Bahrs

**Affiliations:** 1 Department for Traumatology and Reconstructive Surgery, BG Trauma Center, Eberhard Karls University Tübingen, Schnarrenbergstr. 95, 72076 Tübingen, Germany; 2 Clinic for Orthopaedics and Traumatology, St. Josephs-Hospital Wiesbaden, Beethovenstraße 20, 65189 Wiesbaden, Germany

**Keywords:** Constant score, Functional assessment, Proximal humeral fracture, Strength measuring

## Abstract

**Background:**

The Constant score (CS) is one of the most frequently applied tools for the assessment of the shoulder joint. However, evaluation of strength is not standardized leading to potential bias when comparing different studies.

**Methods:**

Seventy-six patients with fractures of the proximal humerus undergoing open reduction and internal fixation (ORIF) were assessed using standardized CS strength measurements at the deltoid muscle insertion and at the wrist in three different arm positions. Variation coefficients were evaluated for each patient and position.

**Results:**

Forty women (57%) and 36 men (43%) were examined 96 months in mean after ORIF. We could state a maximum of 105.3 N difference if measurements were performed at the wrist or the insertion of the deltoid muscle in 90° forward flexion on the injured arm (167.9 ± 83.1 N; 62.6 ± 29.4 N). The lowest variation coefficient of the three performed measurements could be stated at the deltoid muscle insertion in a 90° abduction position in the scapula plane (6.94 ± 5.5).

**Conclusion:**

Following our study results, different positions of force measurement can change the total CS by a whole category (e.g. “very good” to “good”). We recommend performing the measurement at the insertion of the deltoid muscle in a 90° abduction position in the scapula plane. Otherwise, even in the non-injured, it is hard to reach a “normal” shoulder function, based on the CS. When using the CS as outcome parameter, authors must give detailed information about the force measuring and use an exact measuring device.

## Background

Rehabilitation of shoulder function after conservative or operative therapy is essential for patients and the preservation of individual independence. For that reason, numerous scores are used to evaluate post-interventional results. These scores often refer to both, objective measurements and subjective patient perception. Accordingly, one of the most commonly used scores is the Constant Score (CS) [1–3].

The CS was originally designed to assess shoulder disorders in general by combining subjective and objective measurements such as pain (15 points), activities of daily living (20 points), strength (25 points) and the range of motion (40 points) [4]. Different studies showed fair correlation between the CS and other scores evaluating shoulder disorders [5]. The CS is known to provide good inter-rater and intra-rater reliability. However, lack of standardization led to different outcomes, especially regarding force measurement [6]. Therefore, a review of the CS guidelines was released in 2008 [7]. Moreover, a CS protocol was published by Ban et al [8] This protocol proofed fair inter-rater and intra-rater reliability for patients with shoulder impingement and the evaluation of the reliability and agreement of 2 strength devices [9]. Since the subjective evaluation of shoulder function, especially in the elderly patients, often deviate from the objective score result, a relativization of the absolute score is possible by a comparison with age- and gender-specific norms or the contralateral side, which are described by Constant (1986), Yian (2005) and Katolik (2005) [10–12]. Kukkonen et al. showed a minimal clinically relevant difference of 10.4 points using the CS, investigating a patient cohort undergoing rotator cuff repair [13]. However, to the knowledge of the authors, different strength properties and their impact on the CS have never been tested.

The primary aim of this study was to evaluate results of the different arm positions for the force measurements mentioned in several standardized CS protocols [7, 10, 14]. The secondary aim was to show variation coefficients of force measurements and their influence on the general outcome.

## Methods

The study includes data of 191 adult (age > 18) patients who underwent surgical treatment of proximal humeral fractures using fixed angle plate osteosynthesis at the BG Trauma Center - University Hospital Tübingen, Germany. Seventy-six patients were re-examined after surgery and demonstrated bony union (lost to follow up 60.51%). Exclusion criteria for this study were the change of therapeutic concept of an anatomical reconstruction of the humeral head during the follow-up period (e.g. revision surgery with arthroplasty), additional injuries of the shoulder/upper extremity of the ipsi- or contralateral arm, non–shoulder-related severe comorbidities (e.g. dementia) and loss of contact due to death or relocation. All follow-up patients could at least abduct the arm in a 90° position. These results were published by Bahrs et al. in 2015 [15].

The CS is a multi-item 0- to 100-point score (high scores indicate a high level of function) with 10 items, which are half subjectively measured (0 to 35 points) and the other half objectively (0 to 65 points) [7].

**Fig. 1:**
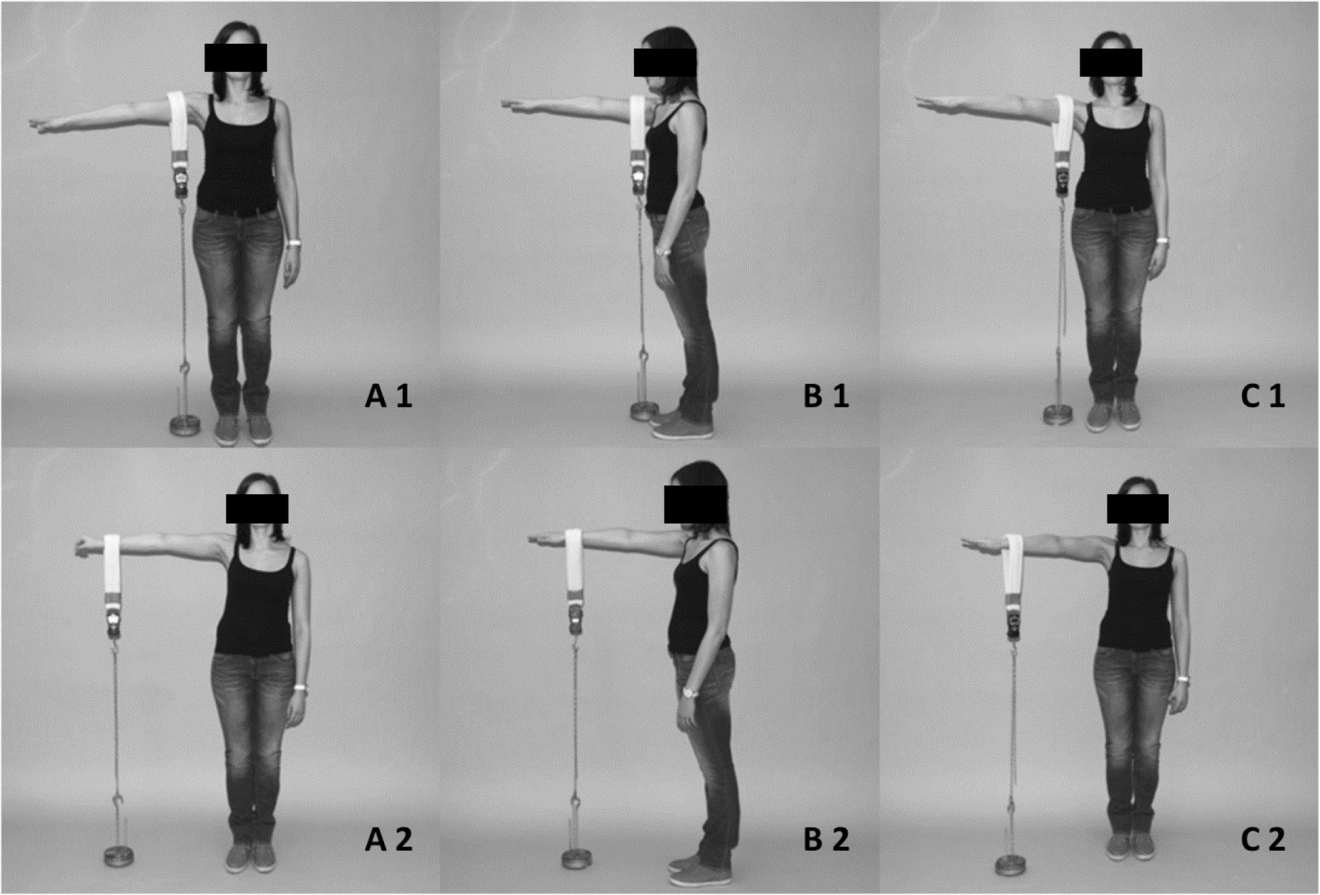
Different testing positions: A1–90 ° abduction deltoid insertion, A2–90 ° abduction wrist, B1–90 ° forward flexion deltoid insertion, B2–90 ° forward flexion wrist, C1–90 ° abduction and 30 ° anteversion deltoid insertion, C2 - 90 ° abduction and 30 ° anteversion deltoid insertion wrist

A score between 86 and 100 points is a “very good” result. A “good” result is considered as a score between 71 and 85 points. Between 56 to 70 points, patients reach a “fair” result and under 56 points the outcome is considered as “poor” [10, 16]. The objective strength part is measured on a continuous scale with a maximum of 25 CS points, whereas the remaining items are rated on an ordinal scale. Following Constant himself, 1 point equals 1 pound of weight (≈0.45 kg), which can be lifted by the arm for 5 seconds [10]. We measured the range of movement of both shoulders using a goniometer during the physical examination. The physical strength was measured by an electronic spring balance (Voltcraft HS-50®, Conrad). The patients had to stand against a wall without leaning against it to assure that they did not make any evasion movement with their torso. Measurements were performed in three different arm positions with two measuring points each (Fig. 1):

Measuring points were the distal humeral insertion of the deltoid muscle and the wrist. The different measuring positions were 90° abduction, followed by 90° abduction / 30° anteversion (scapula plane) and 90° forward flexion of the arm. The elbow was fully extended and the hand in pronated position. Force was measured in Newton (N). Patients had to hold the respective arm over a period of 5 seconds. Testing was performed by a doctoral candidate under supervision of a specialist in orthopedic surgery. Mean values were calculated from three measurements with maximal force and a recovery time of 3 min in between.

In order to elucidate the best measuring arrangement, we calculated variation coefficients for various possible arm positions and measuring points of the non-injured side of each individual patient in any position. The measuring arrangement with the smallest averaged variation coefficient promises the most reproducible measurements and should therefore preferably be used.

Statistical analysis was performed using JMP 10.0.0 (SAS Institute Inc., Cary, North Carolina, USA). Differences between two groups (e.g. men and women) were calculated with the t-test after stating that the data showed normal distribution. The level of significance in the evaluation of the results was recorded with a value of *p* < .05.

## Results

The group included 76 patients, 40 women (53%) and 36 men (47%). Patients were examined at a mean of 96 months (range 74 to 133 months) postoperatively. Mean age was 62 years (range 26 to 90 years) at the time of final follow-up. According to the Neer classification, most of the patients showed 3-part fractures (n = 38, 50%). In 43 patients (57%), plate removal was performed after union at a mean of 11 months after surgery (range 4 to 26 months) (Table 1).

**Table 1:** Demographic data of the included patients

criteria	specification		
total number of patients	[n]	76	
follow up	time [m / range]	92 / 74 – 133 m	
age at time of follow up	total [y / range]	63 / 26 – 90y	
gender	male [n] [% of total]	36	47%
	female [n][% of total]	40	53%
fracture type (Neer Classification)	II part [n]	28	37%
	III part [n]	38	50%
	IV part [n]	10	13%
Implant removal	Yes [n]	43	57%
	No [n]	33	43%

**Fig. 2:**
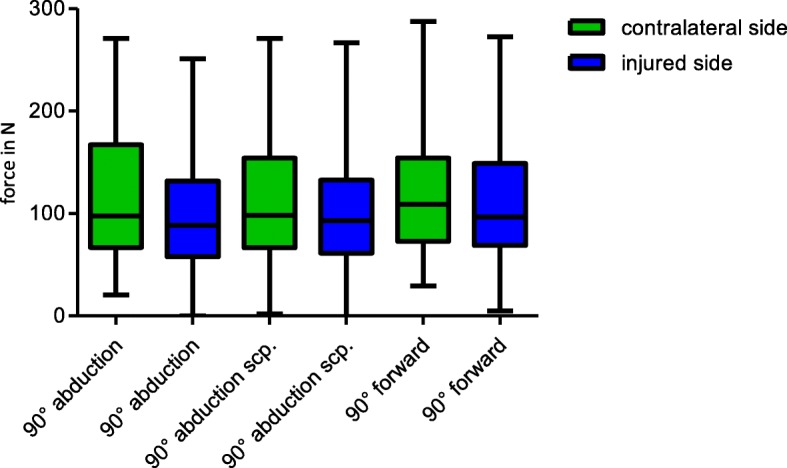
The individual difference between the two measuring points (deltoid insertion and wrist) in Newton (N) for each arm position

Force measurements were performed in all patients of the follow-up group. Patients of the follow –up group could perform a force of 63.8 N (SD ± 31.2 N) on average in 90 ° abduction in the scapula plane, measured at the wrist on the operated side. This represented 82.3% of the strength of the non-injured arm on average (79.0 N; SD ± 36.7 N;). Taking the measurement in the same position but at the insertion of the deltoid muscle we could measure 161.8 N (SD ± 83.5 N) at the injured side (Table 2, Fig. 2). Men showed significantly higher results in all positions than women (*p* = <.0001). Results of force measurements didn’t show a significant difference regarding the impact of the dominant hand. If the injured arm was the dominant arm, patients could reach a force of 83.4 N (SD ± 42.9 N) in 90 ° abduction in the scapula plane at the wrist. If the non-injured arm was the dominant arm, patients showed a force of 82.5 N (SD ± 34.3 N) in mean (*p* = .93).

**Table 2:** Force in N for different positions of measurements

Deltoid								Wrist						
	Contralateral side			Injured side			Diff. in mean	Contralateral side			Injured side			Diff. in mean
position	Mean [N]	SD [N]	CI 95%	Mean [N]	SD [N]	CI 95%		Mean [N]	SD [N]	CI 95%	Mean [N]	SD [N]	CI 95%	
90° abduction	193.1	95.1	171.4–214.8	164.4	85.7	144.8–183.9	28.7 ± 14.7	78.4	37.0	69.9–86.3	64.3	33.3	56.7–71.9	14.1 ± 5.7
90° abduction scapula	189.3	87.7	169.2–209.3	161.8	83.5	142.7–180.9	27.5 ± 13.9	79.0	36.7	70.6–87.3	63.8	31.2	56.7–70.9	15.2 ± 5.5
90° forward flexion	197.9	94.5	176.3–219.5	167.9	83.1	149.0–186.9	30.0 ± 14.4	75.3	32.6	67.8–82.7	62.6	29.4	55.9–69.3	12.7 ± 5.0

Different positions of measuring the force showed very similar results as we compared the reproducibility of three measurements for each position. There was no position with a particularly low variation coefficient (Table 3).

**Table 3:** Variation coefficients of the different measuring points (injured arm)

	Deltoid				Wrist			
position	Mean [N]	SD [N]	Min	Max	Mean [N]	SD [N]	Min	Max
90° abduction	8.37	5.43	0.18	27.73	8.88	7.71	1.11	53.35
90° abduction scapula	6.94	5.50	0.31	21.93	7.98	4.72	1.82	22.72
90° forward flexion	8.18	6.41	1.16	32.05	8.63	6.23	1.23	29.10

**Fig. 3:**
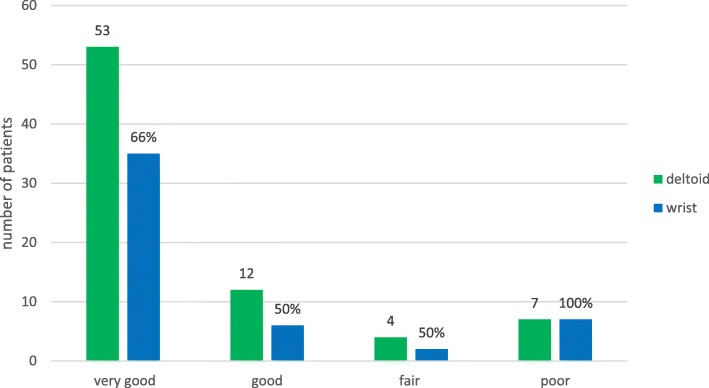
The effect of the different measuring points (deltoid insertion and wrist) on the total Constant Score categorized in “very good”, “good”, “fair” and “poor” outcome. Green bars represent the outcome based on the deltoid measurement for all patients included. Blue bars show a direct comparison of each patient by performing the measurement at the wrist

The effect of different arm positions and measuring points on the CS itself showed a wide variation starting from 63.77 N (± 31.4) to 164.81 N (± 79.5) resulting in a big influence on the score (Fig. 3). These differences can already change a “good” result to a fair result, or a “fair” to a “poor” result in context of the total score. Calculating the total score and categorizing the results, only 66% of the patients with a “very good” result could reach the same category when the measurement was performed at the wrist instead of the deltoid muscle insertion.

As the category “force / strength” is limited to 25 points (25 lb. (=11.34 kg)), the difference within the score between each measurement couldn’t be bigger. The fictional calculated maximum difference would have been 44 points in 90° abduction at the deltoid insertion compared to the wrist as measuring point.

On average, patients showed 79.0 points (± 17.8, median 85 points, 30–100 Points) on the operated side based on the CS. Thirty-six patients (46.8%) achieved a “very good” result (86-100Points), 24 patients (31.2%) a “good” (71–85 points), seven patients (9.1%) showed a “fair” (56–70 points) and ten (13.0%) showed a “poor” result (< 56 points). At the non-injured side an average of 89.6 points (± 9.9; median 90 points; 49–100 points) was stated. Fifty-eight patients (75.3%) showed a “very good”, 15 a “good” (19. 5%), three a “fair” (3.9%) and only one patient (1.3%) a “poor” result.

## Discussion

The aim of our study was to evaluate different results of arm positions and measuring points for force measurements mentioned in several descriptions and publications of the CS. We examined 76 patients with humeral fracture undergoing ORIF. The analyzed data showed a wide range of variation coefficients for the different positions. The achieved force was mainly dependent on whether the measurements were performed at the wrist or the insertion of the deltoid muscle.

The investigation technique for the CS was not exactly defined in the original paper. This could be one reason why there are still numerous problems when different studies are compared [17]. In particular, the individual way of force measurement has a considerable effect on the result. According to Lillkrona, this results in an interobserver variability of 10 at a maximum of 100 achievable points, which is an unsatisfactory condition [18]. Regarding the arm position, most of the authors measure the force only in an abduction position. Others take measurements in anteversion and some in elevation (scapula plane) or don’t use electric spring balances for their measurements which leads to inexact values. In several publications, the measurement method is not clearly defined, which makes the comparison between different studies almost impossible. In order to find the best measurement method, we performed the evaluation of the strength in three different arm positions, each with two different measuring points: 90° abduction, 90° abduction in the scapula plane (30° anteversion) and in 90° forward flexion, each at the distal end of the deltoid muscle and at the wrist.

Variation coefficients for measurement results were, however, very similar for all positions and measuring points and rather high so that no measuring arrangement could be stated as superior in reproducibility and could therefore be particularly recommended.

There is also different information about the measuring point. Constant and Murray et al. favored a measurement at the deltoid muscle insertion [14, 19], whereas Gerber et al. favorited the wrist as measuring point [20]. Comparing the strength of the healthy side in 90° abduction measured in the scapula plane at the wrist with the force defined as normal by Constant (25 lb. (= 11.34 kg)) [10], only 14 patients (22.2%) reached a normal force in our measurements. Yet, as we performed the measurements at the deltoid muscle, 53 patients (80.3%) could reach normal force. Johansson and Adolfsson also concluded, that if force measurements are performed at the wrist, it is difficult even for young and healthy persons to reach 100 points in the CS [21]. Thomas et al. concluded that Constants definition of normal force must refer to the measuring point at the deltoid muscle insertion, since less than 50% of the men and no woman could hold 12 kg in 90° abduction position at the wrist in their investigation [17]. Based on these contradictory indications, the publication by Constant CR et al. is a clarification of the measurement methods [7]. The most important requirements are the force measurement at the wrist and at 90° abduction. For lower abduction, values for the force should be set to zero. This method of measurement, however, leads inevitably to lower values than measurements at the deltoid insertion and at lower abduction.

Balcess-Diaz et al. recently stated, that statistically differences in the CS occur because of age and gender specific differences [22]. Our data showed a lot of variability. We believe, that this can be explained by the wide range of age in our study population. Therefore, for future studies it is necessary to define and describe clearly how the measurement of shoulder force was evaluated. In order to obtain reliable and meaningful score results, especially in comparing alternative therapy methods, the shoulder function should additionally be evaluated with other scores like the DASH or Oxford score, too.

## Conclusion

Based on our study results, we recommend performing the measurement at the insertion of the deltoid muscle in a 90° abduction position in the scapula plane. It is obvious that even for non-injured people, it is hard to reach a “normal” shoulder function, following the CS. When using the CS as outcome parameter, authors must give detailed information about the force measuring and use an exact measuring device.

## Data Availability

The datasets used and analyzed during the current study are available from the corresponding author on reasonable request.

## Abbreviations

CS: Constant Score
kg: kilogram
lb.: libra pondo
ORIF: open reduction and internal fixation
SD: Standard Deviation
